# Practical Electricity in Medicine and Surgery

**Published:** 1890-06

**Authors:** 


**Practical Electricity in Medicine and Surgery.** By G. A. Liebig, Jr., Ph. D., Assistant in Electricity, Johns Hopkins University, etc., and George H. Rohé, M. D., Prof. of Obstetrics and Hygiene, College of Physicians and Surgeons, Baltimore, etc. Profusely illustrated. 8vo, pp. viii, 383. Philadelphia and London: F. A. Davis, publisher. 1890. Price, $2.00.

The authors endeavor to set forth in a concise way the fundamental principles which are involved in the application of electricity to medical and surgical practice. The study of electricity to-day is one of the most important branches of the medical sciences, so widespread and so general has it become in the treatment of disease. It is limited to no special subject as was formerly done, but neurologist, gynecologist, laryngologist, as well as the general practitioner, find it one of the most trusted and reliant therapeutic measures. The authors seem to have grasped its full importance, and have dealt with the subject in a manner at once scientific and comprehensive.

The volume is divided into three parts. In Part I. are discussed the various forms of electrical and magnetic apparatus of use to the physician in his daily experience with electricity, as well as the different arrangement of cells, the construction and use of galvanometers, and a short account of the electric motor, telephone and phonograph as likely to become aids in the diagnosis and treatment of disease. It is almost impossible to treat of these subjects without going into technicalities and mathematics. The authors have simplified, as far as practicable, and have rendered these chapters concise and yet ample.

In Part II. is explained the action of electric currents upon the various tissues of the body in health, then shown how these effects are modified by disease, and finally indicated the methods by which these modifications are utilized for purposes of diagnosis.

In Part III. are considered the applications of electricity in the treatment of diseases. Particular attention is given to the application of electricity in gynecology, diseases of the male genito-urinary organs, and in diseases of the skin.

The illustrations, many of them original, are nicely executed, and add to the usefulness of the book.

The typographical work is up to the standard of this popular publishing house. W. C. K.

